# Extensive myocardial infiltration by hemopoietic precursors in a patient with myelodysplastic syndrome

**DOI:** 10.1186/1471-2326-6-4

**Published:** 2006-09-05

**Authors:** Farrah J Mateen, Sheila R Harding, Anurag Saxena

**Affiliations:** 1 Department of Neurology, Mayo Clinic^1^, Rochester, USA; 2 Departments of Pathology and Internal Medicine, University of Saskatchewan and Saskatoon Health Region, Saskatoon, Canada; 3 Department of Pathology, University of Saskatchewan and Saskatoon Health Region, Saskatoon, Canada

## Abstract

**Background:**

Although myocardial infiltration with leukemic blasts is a known finding in patients with acute leukemia, this phenomenon in myelodysplasia is not reported in the literature. Cardiac symptoms in patients with myelodysplasia are often due to anemia and may be due to iron overload and side effects of therapy.

**Case presentation:**

Herein we report the first case of neoplastic infiltration of the heart with associated myocardial necrosis in a patient with myelodysplasia. It was associated with unicellular and multifocal geographic areas of necrosis in the left ventricle and the interventricular septum. It is likely that cardiac compromise in our patient was due to a combination of restrictive cardiomyopathy due to leukemic infiltration, concomitant anemia, cardiac dilatation, conduction blocks and myocardial necrosis. Myocardial necrosis was most likely due to a combination of ischemic damage secondary to anemia and prolonged hypotension and extensive leukemic infiltration. Markedly rapid decrease in ejection fraction from 66% to 33% also suggests the role of ischemia, since leukemic infiltration is not expected to cause this degree of systolic dysfunction over a 24-hour period. The diagnosis was not suspected during life due to concomitant signs and symptoms of anemia, pulmonary infections, and pericardial and pleural effusions. The patient succumbed to cardiac failure.

**Conclusion:**

Hemopoietic cell infiltration was not considered in the differential diagnosis and contributed to this patient's morbidity and mortality. This case highlights the clinical importance of considering myocardial infiltration in patients with myelodysplasia and cardiac symptoms.

## Background

Myelodysplastic syndromes are hematologic malignancies characterized by dyspoiesis, a progressive clinical course and usually a fatal outcome due to either transformation to acute leukemia or bone marrow failure [1,2]. The patients often present with symptoms attributable to cytopenias and the clinical course reflects progression to bone marrow failure or transformation to acute leukemia [3]. The patients usually managed with supportive care and sometimes novel treatments [4] are monitored for evolution to acute leukemia and progressive marrow failure using some risk stratification scheme, for instance the International Prognostic Scoring System [5].

Cardiac symptoms in patients with myelodysplasia are usually due to anemia [6] or iron overload [7] and sometimes due to toxic effects of drug treatment. The latter include cardiac arrythmias due to a hemopoietic growth factor, IL-11 [8], bradycardia and orthostasis due to immunomodulating thalidomide [9], exacerbation of congestive cardiac failure due to immunomodulating infliximab [10], cytotoxic chemotherapy e.g. cytarabine related cardiotoxicity [11], and conduction abnormalities due to a putative differentiating agent arsenic trioxide [12]. Although infiltration by leukemic blasts is a known phenomenon in patients with acute leukemia [13-15], to the best of our knowledge our's is the first case report of cardiac infiltration by malignant hemopoietic cells in a patient with myelodysplasia. The extensive hemopoietic cell infiltration was not considered in the differential diagnosis and contributed to this patient's morbidity and mortality. This case, therefore, highlights the importance of considering this phenomenon in patients with myelodysplasia who develop cardiac symptoms.

## Case presentation

A 64-year-old woman with increased lethargy, generalized weakness, and shortness of breath on exertion was found to have pancytopenia on a routine blood count; hemoglobin 80 g/L, white blood cells 3.2 × 10^9^/L, platelets 98 × 10^9^/L. After bone marrow examination a diagnosis of refractory anemia with excess blasts (RAEB) was made. The symptoms were attributed to anemia and she received 5 units of packed red cells.

**Figure 1:**
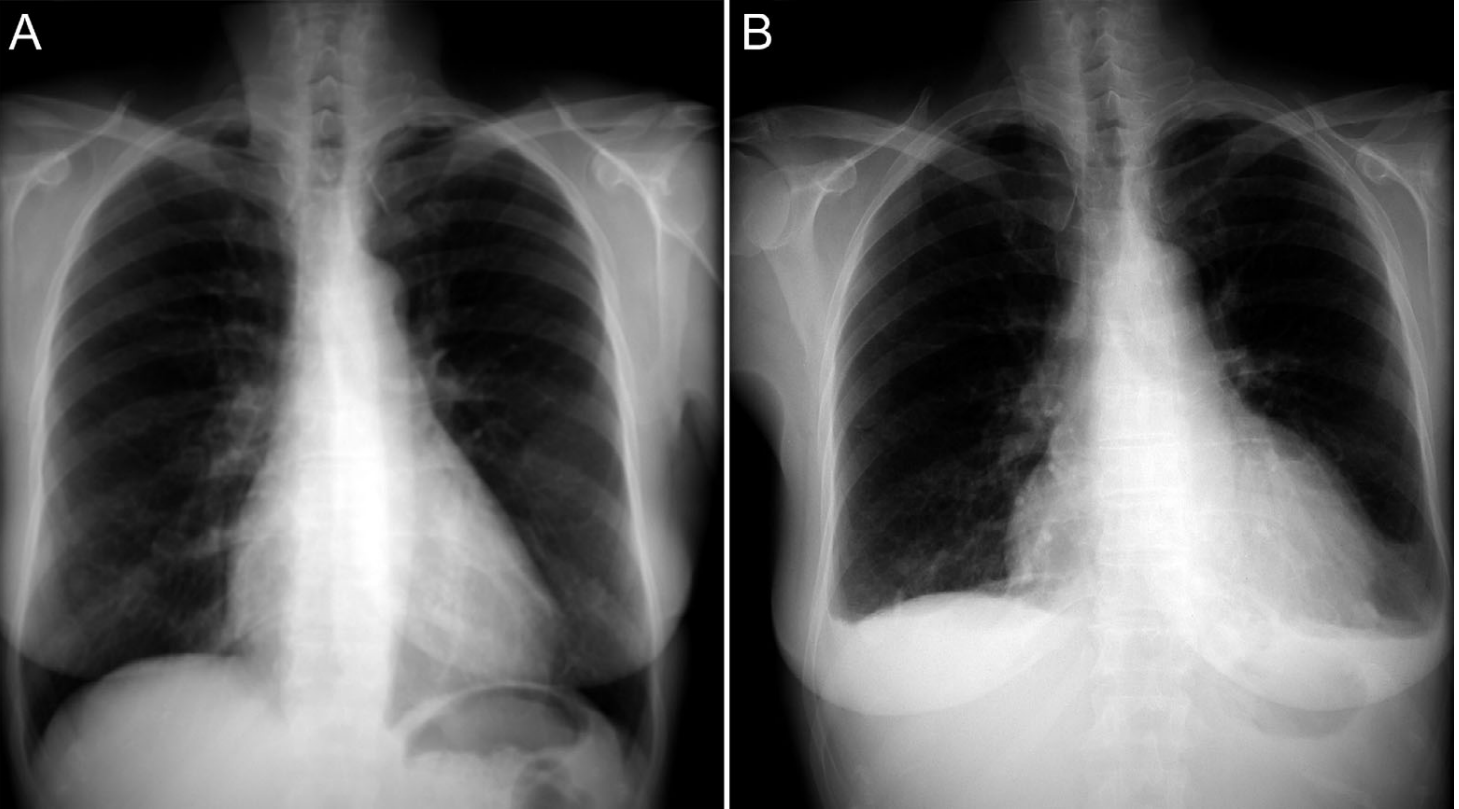
Plain radiographs of the chest (PA views) at admission (a) and four days later (b) with noticeable increase in the cardiac silhouette.

Approximately 2 months later, she developed a 4-day course of intermittent chills and sweating but was afebrile when she came to the local emergency department. The CBC at admission demonstrated 6.9 × 10^9^/L white blood cells with left shift and 0.21 × 10^9^/L blasts, 76 g/L hemoglobin and 65 × 10^9^/L platelets. During her hospital stay the white blood cells increased to 16.9 × 10^9^/L with increasing left shift; anemia and thrombocytopenia persisted. There was central bronchial wall thickening and interstitial prominence in the chest radiograph suggestive of an early viral infectious process. The cardiomediastinal silhouette was within normal limits (Figure 1a). The electrocardiogram (ECG) showed normal sinus rhythm. The patient was started on oral levofloxacin.

Four days later, the patient became febrile (38.8 degrees) and developed increasing shortness of breath and retrosternal chest pain that radiated to both arms. There were bilateral crepitations and decreased breath sounds. Repeat chest radiograph demonstrated bilateral pleural effusions, basal consolidation of the left lower lobe and left ventricular enlargement. There was no evidence of cardiac failure (Figure 1b). Right bundle branch block (RBBB) with sinus tachycardia was identified on ECG. An echocardiographic study identified a moderate pericardial effusion with no cardiac tamponade; the left ventricular ejection fraction was 66% and there were no regional wall motion abnormalities. The next day, a repeat echocardiographic study identified a 33% ejection fraction with left ventricular global hypokinesia and a moderate sized pericardial effusion without tamponade. The patient was treated for pneumonia, hypotension, acute renal failure, and anemia but developed heart block and cardiorespiratory compromise. Her condition deteriorated rapidly and she died five days post-admission.

**Figure 2:**
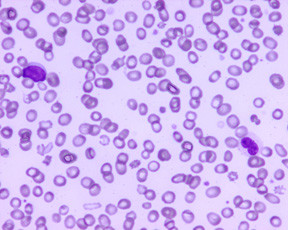
Peripheral blood smear with a circulating blast and a pseudo Pelger-Huet cell, (Giemsa × 500).

**Figure 3:**
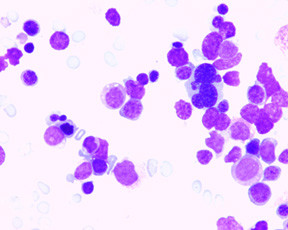
Bone marrow aspirate with marked erythroid dysplasia (Giemsa × 500).

**Figure 4:**
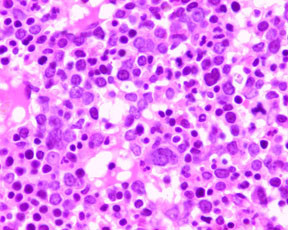
Bone marrow core biopsy with megakaryocytic and erythroid dysplasia (H&E × 300).

**Figure 5:**
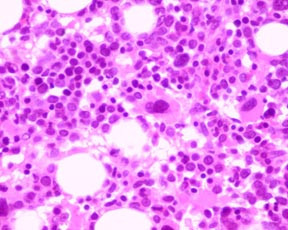
Bone marrow core biopsy with abnormal localization of immature precursors (H&E × 300).

Peripheral blood and bone marrow, ante-mortem (Figures 2, 3, 4, 5): In the peripheral blood there was dysplasia in the leukocytes (pseudo Pelger-Huet cells and hypogranular neutrophils), platelets (hypogranular platelets and large platelets) and red cells (macrocytic red cells and dimorphic red cells) was associated with a left shift and circulating blasts. The bone marrow was hypercellular with trilineage dysplasia (pseudo Pelger-Huet cells, erythroid precursors with nuclear bridging, irregular nuclear contours, irregular hemoglobinization of the cytoplasm and mononuclear and multinucleated megakaryocytes). There was abnormal localization of immature precursors (ALIP) in the core biopsy. The bone marrow aspirate differential count (table 1) showed increased blasts (18.2%) while the erythroid precursors were less than 50% of the nucleated cells.

**Table 1:** Differential count of the bone marrow aspirate and peripheral blood; total peripheral blood leukocyte count = 3.2 × 10^9^/L.

Specimen	Bl	Pr	My	Meta	Band	Gr	Ly	Mo	Ery
Bone marrow (in %)	18.2	19	24.8	6.0	5.0	1.0	4.5	0.5	21
Peripheral blood (in absolute count, × 10^9^/L)	0.03	0.13	0.11	0	0.19	0.35	2.36	0.03	No nucleated erythroid precursors

Bl: Blasts, Pr: Promyelocytes, My: Myelocytes, Meta: Metamyelocytes, Band: band forms, Gr: Granulocytes, Ly; Lymphocytes, Mo: Monocytes, Ery: Erythroid precursors

Post-mortem examination was limited to heart and lungs at the request of the family. The heart lay free in the pericardial sac, surrounded by 300 mL of straw-colored pericardial effusion. There was fibrinous pericarditis. The free wall of the left ventricle and the interventricular septum had soft and hemorrhagic areas scattered throughout, with no definite transmural focus. The major coronary arteries (right coronary, left anterior descending, and left circumflex arteries) were involved to only a minor degree by old eccentric atherosclerotic plaques (maximum stenosis of 25 to 30%) with no evidence of an acute event (thromboembolus, hemorrhage, rupture). There was bilateral pulmonary edema and left lower lobe congestion and consolidation.

**Figure 6:**
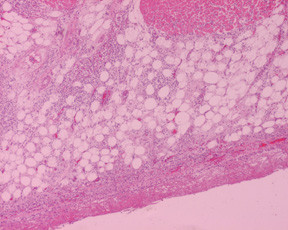
Epicardial involvement by hemopoietic precursors with fibrinous pericarditis (H&E × 50).

**Figure 7:**
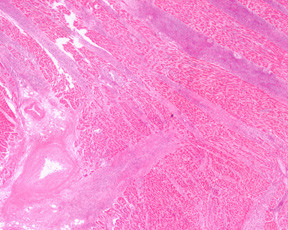
Extensive septal and intercellular myocardial infiltration with vascular wall andmyocardial necrosis (H&E × 50).

**Figure 8:**
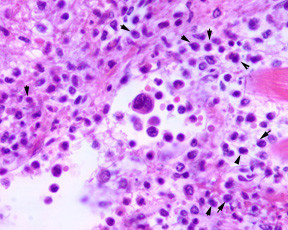
Cardiac infiltration by hemopoietic precursors; dysplastic megakaryocyte andmyeloid precursors; arrows point to blasts (H&E × 300).

**Figure 9:**
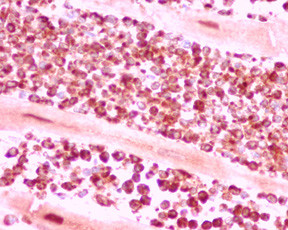
Cardiac infiltration by hemopoietic precursors; Myeloperoxidase positive myeloidprecursors (Immunoperoxidase × 300).

Microscopic examination of the heart (Figures 6, 7, 8, 9) revealed a diffuse interstitial infiltrate of immature dysplastic hemopoietic cells involving the myocardium, endocardium and the pericardium. Cells of myeloid, erythroid and megakaryocytic lineages were present. These infiltrates were associated with single fibre myocyte necrosis as well as larger foci of necrosis. A majority of these cells were immunopositive for myeloperoxidase consisted with myeloid lineage.

Pancytopenia associated with peripheral blood features including dysplastic changes in the granulocytes, <1% blasts and < 1 × 10^9^/L monocytes coupled with multilineage dysplasia and 18.2% blasts in the bone marrow was in keeping with the diagnosis of refractory anemia with excess blasts-2 (RAEB-2) [1]. Clinical presentation and clinical course of RAEB is typically related to the symptoms of decreased counts of one or more cell lineages and blast count [3,4]. The symptoms of lethargy, shortness of breath, and generalized weakness in this patient may be attributed to both anemia and cardiac infiltration by malignant cells, although the former is much more common clinically [6]. Her cardiac symptoms were not due to drugs sometimes used in patients with myelodysplasia, since the patient had not received cytotoxic, immunomodulatory, putative differentiating agent, or hemopoietic growth factor therapy [8-12].

Although cardiac involvement in leukemic extramedullary spread is relatively common (ranging from 37–44%) [13-15], clinical signs are found in less than 1% of cases [16-18] and leukemic cardiac involvement antemortem is usually not suspected [19]. The most likely reason for this is the subclinical nature of the symptoms and signs in cardiac leukemic infiltration [20]. This is in keeping with the observation that gross infiltrative disease at the time of initial diagnosis in patients with acute leukemia is rare [14,19,21]. In the patient reported here extramedullary (cardiac) infiltration by dysplastic hemopoietic precursors by itself is not a reason to upgrade the diagnosis to acute leukemia. Extramedullary infiltration at other sites has been reported in patients with myelodysplastic syndromes [22,23], more frequently in patients with chronic myelomonocytic leukemia compared to RAEB and refractory cytopenia with multilineage dysplasia [23,24]. Although it may herald transformation to acute leukemia [25], this transformation may not be observed for some time [26,27] and sometimes not at all during follow-up [28,29]. Granulocyte-macrophage colony stimulating factor overproduction may lead to autonomous colony formation in the bone marrow of patients with myelodysplastic syndrome [30]; this may partially explain proliferation of malignant hemopoietic cells in the heart infiltrated by malignant cells.

Although cardiac infiltration is usually associated with high WBC count (mostly due to blasts) and advanced disease [14], the presence of a high circulating white blood cell count is not a necessity for developing cardiac infiltration as infiltration has been shown to be present in aleukemic leukemia [21] as well as in patients with very low white cell counts [31]. The development of cardiac infiltration in our patient with myelodysplasia and pancytopenia would be consistent with this observation keeping with this pheneomenon; the rising white late in the course of the disease was was predominantly due to neutrophilia and left shift and not due to a large blast population.

The effects of hemopoietic cell infiltrate in the heart are varied. Leukemic deposits may form mass lesions [32] or thrombi [33]. Pericardial involvement may lead to pericardial effusion contributing to restrictive myocardial dysfunction [19,34]. Reports of heart block in extramedullary cardiac leukemic involvement are few [31,35,36]. Heart block has been observed in patients with both very high and very low peripheral blood white cell counts [31] and may be reversible after local radiotherapy to the heart despite persistence of leukemic infiltration [31]. However, infiltration of the conduction system is a potentially serious complication that may be fatal [37]. Leukemic infiltration is a rare cause of restrictive cardiomyopathy [18]. An antemortem study of 18 patients with acute leukemia (6 ALL, 12 AML) demonstrated no significant difference from controls in LV systolic function parameters including LV ejection fraction, similar to what was observed in our patient at initial echocardiography [18]. However, LV diastolic dysfunction has been observed in 38 percent of leukemic patients, independent of age and heart rate. It is likely that cardiac compromise in our patient was due to a combination of restrictive cardiomyopathy due to leukemic infiltration, concomitant anemia, cardiac dilatation, conduction blocks and myocardial necrosis. Myocardial necrosis was most likely due to a combination of, a) ischemic damage secondary to anemia and prolonged hypotension and b) extensive leukemic infiltration. Markedly rapid decrease in ejection fraction from 66% to 33% also suggests the role of ischemia, since leukemic infiltration is not expected to cause this degree of systolic dysfunction over a 24-hour period.

Usual causes of death in patients with myelodysplasia are related to bone marrow failure and transformation to acute leukemia [3,4,38], however, in this patient, death was attributed to cardiac failure. It is likely that the rising white blood cell count during second admission, although predominantly due to neutrophilia and left shift, was associated with early transformation- in view of increased peripheral blood blast percentage – the limited autopsy did not permit evaluation of the bone marrow.

Cardiac involvement in by malignant hemopoietic cells is of more than just academic interest, since cardiac function has been shown to improve following therapy directed against malignant infiltrate [31,39]. Incorrect diagnosis during life and the fatal outcome highlight the clinical importance of considering myocardial infiltration in patients with myelodysplasia and cardiac symptoms.

## Conclusion

Infiltration of heart tissues by malignant hemopoietic cells can occur in patients with myelodysplasia. This case highlights that in a patient with myelodysplasia, leukemic cardiac infiltration should be considered in the differential diagnoses when investigating cardiac symptoms and signs, particularly heart block.

## Competing interests

The author(s) declare that they have no competing interests.

## Authors' contributions

SRH was responsible for the initial assessment and management of the patient. AS was responsible for the initial diagnosis of myelodysplasia and also performed the autopsy on the deceased. FM was responsible for review of the patient's clinical charts and interpretation of data. All authors have contributed equally in the preparation of the manuscript. All authors read and approved the final manuscript.

## Pre-publication history

The pre-publication history for this paper can be accessed here:


